# Assessing trends of breast cancer and carcinoma *in situ* to monitor screening policies in developing settings

**DOI:** 10.1038/s41598-019-50504-6

**Published:** 2019-10-02

**Authors:** Érika de Abreu Costa Brito, Marcela Sampaio Lima, Hianga Fayssa Fernandes Siqueira, Adriane Dórea Marques, Alex Rodrigues Moura, Evânia Curvelo Hora, Carlos Anselmo Lima, Marceli de Oliveira Santos, Mirian Carvalho de Souza, Angela Maria da Silva, Hugo Leite de Farias Brito, Rosana Cipolotti

**Affiliations:** 1 Aracaju Cancer Registry, Aracaju, Sergipe, Brazil; 2 Health Sciences Graduate Program, Aracaju, Sergipe, Brazil; 3 University Hospital/EBSERH/Federal University of Sergipe, Aracaju, Sergipe, Brazil; 4 CONPREV/Brazilian National Cancer Institute, Rio de Janeiro, Brazil; 5 Researcher, CHAMADA MS/CNPq/FAPITEC/SE/SES – No 06/2018, Sergipe, Brazil

**Keywords:** Health policy, Cancer epidemiology

## Abstract

There have been arguments about the role of breast cancer screening at the population level, and some points of controversy have arisen, such the establishment of organized screening policies and the age at which to begin screening. The real benefit of screening has been questioned because the results of this practice may increase the diagnosis of indolent lesions without decreasing mortality due to breast cancer. The authors have proposed a study of incidence and mortality trends for breast cancer in a developing setting in Brazil to monitor the effectiveness of the official recommendations that prioritize the age group from 50 to 69 years. The database of the Cancer Registry and the Mortality Information System was used to calculate age-standardized and age-specific rates, which were then used to calculate incidence and mortality trends using the Joinpoint Regression Program. The results showed stability in trends across all ages and age-specific groups in both incidence and mortality. In conclusion, we found that incidence and mortality rates are compatible with those in regions with similar human development indexes, and trends have demonstrated stabilization. Thus, we do not endorse changes in the official recommendations to conduct screening for ages other than 50 to 69 years, nor should policy makers implement organized screening strategies.

## Introduction

Considering the epidemiological transition of developing countries, breast cancer has become a growing burden in these areas^1^. Brazil has been experiencing increasing incidence rates, especially in state capitals and more developed regions^2^. Mortality rates have also remained high^3^. Breast cancer is the type of cancer with the highest mortality rate among women.

Brazilian cancer registries cover less than 50% of the Brazilian population, and incidence rates are obtained by estimates made by the Brazilian National Cancer Institute (INCA) every two years. It has been estimated that the mean age-standardized incidence rate for 2018–2019 in Brazil is 51.3 per 100,000 women; in the state capitals, the estimated rate is 64.0 per 100,000 women^4^. Information regarding the impact of mortality comes from the analysis of the database of the Mortality Information System (SIM). For 2016, the age-standardized mortality rate in Brazil was 12.7 per 100,000 women^5^.

The role of screening has been discussed, including whether it has actually been effective in decreasing mortality and not just increasing survival and whether the difference is due only to the overdiagnosis of approximately 30% additional cases obtained by screening mammography^6–8^.

A major point of argument regarding screening has been the age at which to start. Many organizations associated with cancer control have advocated starting screening at the age of 40 years, emphasizing the associated increase in survival. However, we must consider the possibility of diagnosing indolent lesions, which inflates the incidence statistics and leads to consequent overtreatment that could be harmful to the patient^9–11^.

In Brazil, there is no policy for systematic screening; instead, there are recommendations, and the Ministry of Health has stated that for the general population, the age group of 50 to 69 years should be prioritized for mammographic screening every two years^12^.

Thus, we have decided to carry out a study of trends in the incidence and mortality of female breast cancer, as opposed to an analysis of the trend in carcinoma *in situ*, to monitor the effectiveness of the official recommendations for the breast cancer screening in a controlled population of a capital in northeastern Brazil with a high-quality cancer registry.

## Methods

The municipality of Aracaju, capital of the state of Sergipe, Brazil, is located at 10°54 ′36″S, 37°4′12″ W and was the site considered for this study. Its Human Development Index (HDI) is 0.770, and the estimated population in 2018 was 648,939 inhabitants^13,14^.

Incidence data were obtained from the database of the Aracaju Cancer Registry from 1998 to 2014; mortality data were collected from the Mortality Information System (SIM) for the same period.

The collection of cancer cases by the Cancer Registry follows the recommendations of the IARC^15,16^, which were validated in Brazil by INCA. The Cancer Registry data are nationally validated and have been used to calculate estimates of cancer incidence in Brazil^4^ and internationally, through their use in the publications of the IARC and the CONCORD Working Group^17,18^.

We analyzed all cases of invasive breast cancer and pure carcinoma *in situ* and all deaths associated with breast cancer occurring within the defined period using codes C50 and D05 of the International Classification of Diseases (ICD), 10th revision. We included all cases with the following morphological codes, ICD-Oncology, 3^rd^ revision: 8000 (undefined morphology), 8022, 8035, 8200, 8201, 8211, 8246, 8401, 8470, 8480, 8500, 8503, 8504, 8510, 8520, 8530, 8540, 8575, 8850, 8890, 8900, 9020, and 9120.

To calculate the rates, we used the census population and the population estimates for the study area provided by the Brazilian Institute of Geography and Statistics (IBGE)^14^. Subsequently, we calculated the age-standardized rates, taking as reference the world population^19^, and their respective confidence intervals for each year. We also calculated the age-specific rates with five-year intervals according to the standard for the Cancer Registry and for the study; for the best evaluation of the screening recommendations, we reorganized the age groups as follows: 20 to 39, 40 to 49, 50 to 69, and 70+ years. For carcinoma *in situ*, due to the lower number of cases, we divided the patients into age groups of <50 and 50+ years, as seen in the supplementary file.

The Joinpoint Regression Program, version 4.6.0.0, from the National Cancer Institute, USA^20^, was used to obtain the trends. The calculations were made by using the Grid Search Method, the program’s default, which defines a minimum of zero to a maximum of three joinpoints. The permutation test was chosen for the model selection, using a significance level of 0.05 and maximum permutations of 4499. Then, the annual percentage change (APC) and the average annual percentage change (AAPC) and their confidence intervals were defined by the parametric method^21^. We also calculated the mortality-to-incidence rate with confidence intervals and its complement 1-(M/I) to infer annual survival.

This project was approved by the Ethics and Research Committee of the Federal University of Sergipe under CAAE number: 57995416.9.0000.5546. We reiterate that all methods were performed in accordance with the relevant guidelines and regulations. To maintain confidentiality, we used deidentified databases, for which it would have been impracticable to obtain informed consent. Thus, according to Resolution 466 of December 2012 of the Ministry of Health, Brazil, we asked the Ethics Committee for exemption of the informed consent, which was granted.

## Results

Data collected from the Cancer Registry database from 1998 to 2014 yielded 2,569 cases of invasive carcinoma and 154 cases of carcinoma *in situ*; 759 deaths were extracted from the SIM. In Table 1, we show the distribution by age group. Table 2 shows the number of annual cases and age-standardized rates with their confidence intervals for invasive carcinoma, carcinoma *in situ* and deaths.

**Table 1:** Number and percentage (%) of cases, age-standardized rates for all ages, age-specific rates distributed by age groups, incidence of invasive breast carcinoma and carcinoma *in situ*, and deaths, 1998–2014.

Age group	Invasive	%	Rate	*In situ*	%	Rate	Death	%	Rate
20–39	285	11.1	18.2	12	7.8	1.7	68	9.0	4.6
40–49	623	24.3	105.4	47	30.5	7.1	148	19.5	24.1
50–69	1133	44.1	186.6	68	44.2	9.1	317	41.8	52.4
70+	528	20.5	268.3	27	17.5	11.8	226	29.8	121.7
All	2569	100.0	56.8	154	100.0	2.9	759	100.0	16.2

**Table 2:** Data on number of cases (N), annual age-standardized rates of invasive breast carcinoma, carcinoma *in situ*, and mortality with confidence intervals; mortality-to-incidence ratios (M/I) with confidence intervals, 1998–2014.

Year	Incidence, invasive			Incidence, *in situ*			Mortality			M/I ratio	
	N(2569)	ASR	95% CI	N(154)	ASR	95% CI	N(759)	ASR	95% CI	M/I	95% CI
1998	91	51.4	40.8; 62.0	2	1.1	−0.4; 2.6	24	13.6	8.1; 19.0	0.3	0.2; 0.5
1999	104	55.7	45.0; 66.4	2	0.8	−0.3; 2.0	31	15.7	10.1; 21.2	0.3	0.2; 0.5
2000	115	53.5	43.7; 63.3	1	0.5	−0.5; 1.5	33	14.9	9.8; 20.0	0.3	0.2; 0.5
2001	117	56.5	46.2; 66.7	1	0.5	−0.4; 1.4	49	22.6	16.3; 28.9	0.4	0.3; 0.6
2002	141	63.5	53.0; 74.0	1	0.5	−0.4; 1.3	25	10.9	6.6; 15.1	0.2	0.1; 0.3
2003	110	51.0	41.4; 60.5	3	1.7	−0.2; 3.5	43	21.1	14.8; 27.4	0.4	0.3; 0.7
2004	135	61.1	50.8; 71.4	6	2.7	0.5; 4.9	25	11.5	7.0; 16.0	0.2	0,1; 0.3
2005	147	65.2	54.6; 75.7	4	1.8	0.0; 3.6	47	20.4	14.5; 26.2	0.3	0.2; 0.5
2006	144	63.2	52.8; 73.5	6	2.2	0.4; 4.0	36	15.6	10.5; 20.8	0.2	0.1; 0.4
2007	154	57.3	48.2; 66.4	9	2.9	1.0; 4.8	35	12.6	8.4; 16.7	0.2	0.1; 0.4
2008	159	54.6	46.1; 63.1	11	4.1	1.7; 6.5	51	17.9	13.0; 22.8	0.3	0.2; 0.5
2009	176	60.4	51.4; 69.3	16	5.3	2.7; 7.9	54	17.9	13.1; 22.7	0.3	0.2; 0.5
2010	188	58.2	49.9; 66.5	12	3.8	1.7; 6.0	57	17.5	12.9; 22.0	0.3	0.2; 0.5
2011	203	62.8	54.2; 71.5	9	2.5	0.9; 4.1	51	15.1	10.9; 19.2	0.2	0.1; 0.4
2012	195	59.2	50.9; 67.5	17	5.1	2.6; 7.5	68	19.5	14.9; 24.2	0.3	0.2; 0.5
2013	179	54.3	46.4; 62.3	27	7.7	4.8; 10.6	65	19.5	14.8; 24.3	0.4	0.2; 0.6
2014	211	59.0	51.0; 66.9	27	7.7	4.8; 10.6	65	18.3	13.9; 22.8	0.3	0.2; 0.5

**Figure 1:**
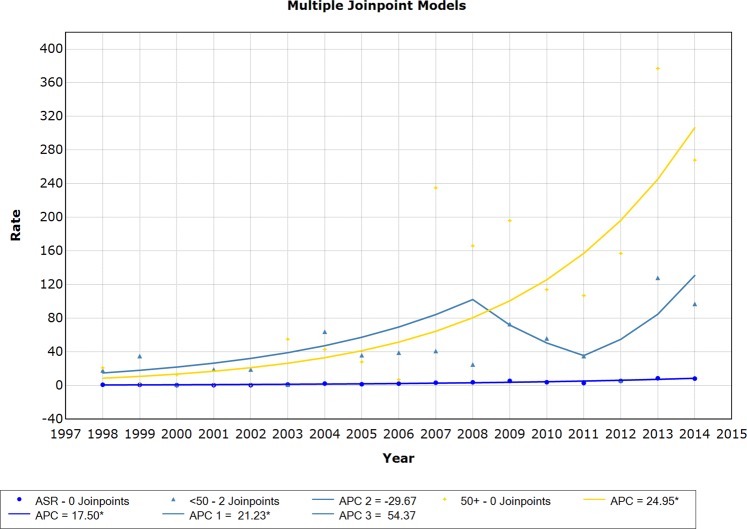
Incidence trends for breast carcinoma *in situ* considering age-standardized rates (ASR) and age groups <50 years and 50+ years, 1998–2014.

For carcinoma *in situ*, due to the small number of cases, the incidence trends are presented according to age groups: under 50 years of age and more than 50 years of age. Thus, we observed growing trends for all age groups, but mainly due to a greater increase in the number of women over 50 years of age, as shown in Fig. 1. In women below 50 years of age, the first period, from 1998 to 2008, displayed a statistically significant APC of 21.2. The second (2008 to 2011) and third (2011 to 2014) periods showed nonsignificant trends (APC −29.67 and APC 54.37, respectively). The increasing trend in the first period might be due to the progressive increase in the use of mammography as a screening method. The diagnosis of carcinoma *in situ*, despite increasing over the time series, was only incipient and was not sufficient to decrease the rates of invasive neoplasms.

**Figure 2:**
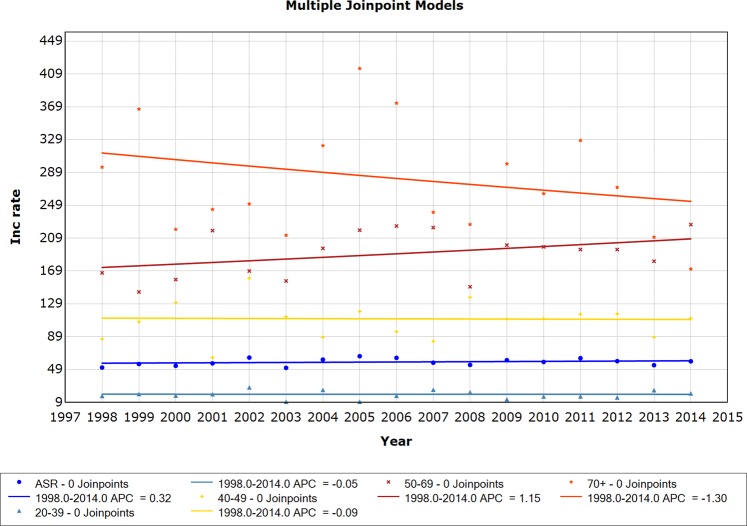
Incidence trends for invasive breast carcinoma, considering age-standardized rates (ASR) and age groups 20–39, 40–49, 50–59 and 70+ years, 1998–2014.

Breast cancer incidence trends remained stable throughout the time series for all ages and defined age groups. The age group 50–69 years showed an upward but nonsignificant trend (APC 1.1), and the age group 70+ years showed a downward and nonsignificant trend (APC −1.3). (Fig. 2).

**Figure 3:**
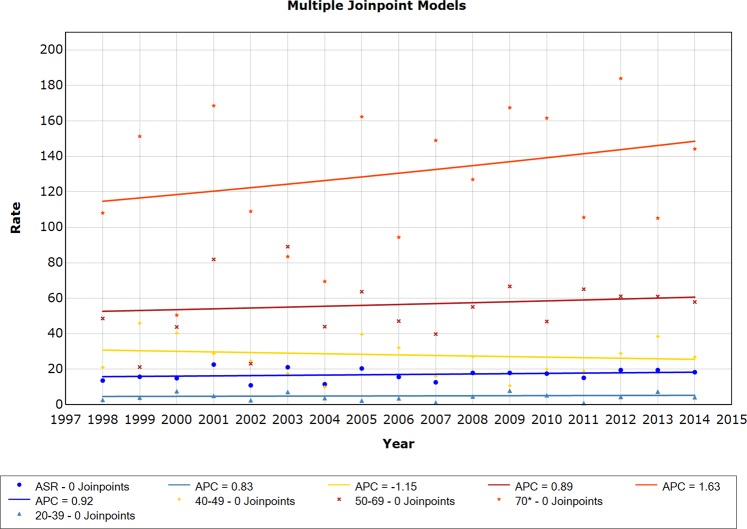
Mortality trends for breast cancer considering age-standardized rates (ASR) and age groups 20–39, 40–49, 50–59 and 70+ years, 1998–2014.

Regarding mortality, none of the age groups demonstrated statistically significant trends. Figure 3 depicts trend showing that the 40- to 49-year age group presented a downward trend (APC −1.15), and the age groups 50–69 and 70+ years exhibited upward trends (APC 0.89 and 1.63, respectively).

In Table 2, we also present the mortality-to-incidence ratios (M/I) and their confidence intervals for each year of the series that. As a proxy of the 5-year survival rate by the complement [1 − (M/I)] (Refs), these values presents an average survival of 70% for the studied population.

## Discussion

This study used a long time series for the analysis of breast cancer trends. The division of the study population into the proposed age groups allowed us to analyze the evolution of incidence rates and to check the effectiveness of the recommended screening policy in Brazil, which starts at age 50 years for the general population, in contrast to the opinions of some cancer control organizations that claim that population screening should begin at the age of 40 years^11,12,22^.

We analyzed the databases of the Aracaju Cancer Registry and SIM, and after calculating the age-standardized and age-specific rates of breast cancer incidence and mortality, we noticed that trends remained stable throughout the time series. Because there has been no systematic screening, we have noticed that a very small number of cases of carcinoma *in situ* was diagnosed, but the number shows an increasing trend.

Whether breast cancer screening results in an earlier diagnosis that improves prognosis or simply contributes to a rise in incidence without decreasing mortality is still a matter of debate. North American and European studies indicate that the 50–69 year age group is the one that benefits from screening practices^11,22,23^. Some medical organizations in those developed countries have started to revise their guidelines accordingly because the increased incidence of breast cancer due to early diagnosis has not had a significant impact on the reduction of mortality.

Screening guidelines are based on the systematic application of tests in a healthy population with the aim of identifying diseases in their preclinical (asymptomatic) stages. The target population is invited/called to participate in screenings that include periodicity, monitoring and evaluation during all stages of the process. The application of screening tests should be capable of reducing mortality from diseases of interest. Conversely, the recommendations are for nonsystematic screening strategies that do not involve the active recruitment of the target population. Opportunistic screening is performed when an individual seeks health services for other reasons, including awareness of the importance of periodic medical examinations^12^.

In Brazil, there is no organized screening policy. Instead, there have been some campaigns, such as “Outubro Rosa” (Pink October), mass awareness programs regarding breast self-examination, and the disbursal of mobile mammography units to remote areas. Evidence-based guidelines on early detection have been published periodically and have guided health professionals in their daily practice and helped to provide greater efficiency in the allocation of resources to benefit the target population, thus minimizing damage. The Ministry of Health has recommended prioritizing women aged 50 to 69 years for mammographic screening every two years. However, opportunistic and sometimes organized screening of women under 50 years of age, has also occurred, contrary to official recommendations^23^.

Screening has increased the incidence of carcinoma *in situ* and indolent lesions^24^, which in some cases account for almost 20% of cases of breast carcinoma^25^. Controversies exist regarding whether the diagnosis of initial lesions by mammography reduces the occurrence of more advanced lesions, consequently decreasing mortality^26^. The decrease in mortality, however, has been due to advances in the treatment of cancer, mainly due to the personalized approach, and not necessarily to mammographic screening^27^. Studies have shown that the most striking effect of screening is overdiagnosis^28,29^. Our data, however, do not allow us to infer whether women from any of the analyzed age groups were more likely to participate in therapies after screening programs.

Da Costa *et al*.^23^ demonstrated an increase in the incidence of carcinoma *in situ*, with incidence rates much higher than those found in our study, in the Regional Health District of Barretos, SP, Brazil. This turns out to reflect the policy of mass screening, including the use of mobile mammography, conducted in the region by a cancer-fighting institution. The increasing trends in the incidence rates of both carcinoma *in situ* and invasive carcinoma have not led to decreased mortality rates. The question is, the use of organized screening policies, including screening for women under 50 years, has benefitted the population at an average risk? On the other hand, it is known that such screening practices have inflated cancer statistics, and harms consequent to overtreatment may ensue.

In our study, we used mean incidence and mortality rates of 58.1 per 100,000 and 16.7 per 100,000, respectively, to calculate the mortality-to-incidence ratio. Using this ratio as a proxy for five-year survival, we obtained an estimate of survival of 71%.

Breast cancer incidence varies according to geographic regions and their HDIs. Hereditary and genetic factors, such as a personal or family history of breast cancer and some inherited mutations, BRCA1 and BRCA2, are important predictors of the development of breast cancer. However, nonhereditary factors, usually associated with Western lifestyle (nulliparity, late age at first birth, fewer children, oral contraceptive use, hormone replacement therapy, and greater weight, among others), play a major role in the development of breast cancer in countries with higher HDIs^30,31^. According to the 2018 Globocan project, breast cancer incidence rates have increased in most developing countries facing epidemiological transition, such as countries in South America, Africa and Asia^30^.

Our data exhibited mean incidence and mortality rates of 56.8 and 16.2 per 100,000 women. According to the 2018 Globocan project, these data are similar to those observed in countries with similar HDIs; incidence rates of 56.8 in South America, 54.5 in Eastern Europe and 50.2 per 100,000 women in the Caribbean and mortality rates of 13.4 in South America, 15.5 in Eastern Europe and 18.1 per 100,000 women in Caribbean have been reported^30^. A study by Ferlay *et al*. demonstrated a variation in breast cancer incidence from 60 to 155 per 100,000 women from eastern Europe to the northern areas, respectively. Mortality rates, on the other hand, ranged from 15 to 32 per 100,000 women from less to more developed areas^32,33^.

Analyses of population survival are excellent strategies for evaluating the effectiveness of health services for cancer control. In addition, data quality assessment allows verification of whether control programs are efficient not only for reducing mortality but also for increasing survival^18^. Differences in survival that disfavor less-developed areas have been observed^34^. Therefore, measures to improve health services need to be implemented to provide equity in cancer control.

The 5-year survival for breast cancer was assessed by the CONCORD-3 study and calculated by the Pohar-Perme estimator using data from six Brazilian cancer registries for the period 2010 to 2014. A 5-year survival rate of 72.5% was observed. This is similar to the survival rate in our study estimated by the complement of the mortality-to-incidence ratio, which is also comparable to the survival rates observed in other regions with equal HDI^18,23^. Reis *et al*. also reported the estimated 5-year survival by using the complement of the mortality-to-incidence ratio; analyzing different series over five years, they found survival rates of 68%, 73%, 78%, 79%, and 72% for the north, northeast, middle-west, southwest, and north regions of Brazil, respectively^35^. The improvement of the quality of mortality information in Brazil as well as the incidence data, such as those in the present study, has allowed a more reliable evaluation of cancer control policies in the study area.

Steponavicine *et al*.^26^ have demonstrated through a time series from 1998 to 2012 that there has been an increased detection of early lesions and reduction of more advanced tumors; however, the increase in the diagnosis of early lesions has been less pronounced since the implementation of the Mass Screening Program in 2006. For the more advanced lesions, incidence trends have remained stable since the initiation of the program. The analysis, however, would have been more precise if the authors had included mortality trends for the same time period.

Analyzing trends for the proposed age groups, we found that incidence trends have remained stable in all groups. Mortality trends have also maintained stability, compatible with other studies from areas with equal socioeconomic development^3,23,36^. Therefore, investing in mass screening policies, as well as including age groups other than 50 to 69 years of age, may not be justified. We do not mean that screening is not necessary; instead, health policies should focus on the specific age groups that could benefit the most. High-resolution mammography has been overused so far, with the consequent overdiagnosis of precursor and indolent lesions that do not necessarily need to be treated. This has led to high health expenditures. In addition, after the detection of indolent tumors, women have become apprehensive and have seemed to have a reduced quality of life because of concern about disease progression and treatment.

Breast cancer awareness campaigns should be encouraged. Information on risk factors should be spread to identify women who need special orientation for their screening. Access to diagnostic and treatment services should be facilitated to avoid delays in the management of cases. As a personalized approach is considered to be the key factor in decreasing mortality and increasing survival, government policies should better target their financial resources to more effectively combat breast cancer.

This research presents strengths related to the good quality of the cancer registry database and the improvement in the quality of mortality information in Brazil. The main limitations are the lack of analysis by histologic subtype and staging of breast cancer. To respond to the latter, our group has been conducting a cohort study that aims to correlate histological subtypes and staging of breast cancer with survival. The Aracaju Cancer Registry has not collected staging information, but it has been improving the quality of its data to continue participating in international publications. The failure to assess actual survival calculation, for example, by the Pohar-Perme estimator, may be a limitation, but the use of the complement of the mortality-to-incidence ratio may provide a reasonable estimate, although this method has been criticized by some^37^.

We did not assess differences in the incidence of breast cancer among races. Race is not a good parameter in Brazil because of the high level of miscegenation, especially in the northeastern region, and because there is no national consensus on identification by race. The classification is based on the race the individual claims to be and usually reflects some social prejudices. Thus, in our opinion, any attempted classification by race is unreliable.

## Conclusions

The incidence and mortality rates of female breast cancer in this developing area of northeastern Brazil display incidence and mortality rates that are similar to those of median risk areas, that is, they are compatible with other regions with similar levels of human development. We recommend that screening policies be reserved for the age group of 50–69 years because the increased incidence of breast cancer due to early diagnosis has not caused a significant impact on the reduction of mortality. Some publications corroborate this recommendation, signaling that this age group should probably benefit the most and that a further decrease in mortality might follow personalized breast cancer treatment. Thus, we do not endorse changes in the Ministry of Health’s recommendations that prioritize the age group from 50 to 69 years to mammographic screening, nor should organized screening practices be adopted. Even so, breast cancer awareness measures should be implemented, and the female population should have optimal access to diagnostic and treatment services.

## Supplementary information

Assessing trends of breast cancer and carcinoma in situ to monitor screening policies in developing settings
