# Supplementary material for: Assessing trends of breast cancer and carcinoma in situ to monitor screening policies in developing settings
Source: Sci Rep. 2019 Oct 2;9:14144. doi: 10.1038/s41598-019-50504-6 (PMC6775160; doi:10.1038/s41598-019-50504-6)

Assessing trends of breast cancer and carcinoma in situ to monitor screening policies in developing settings

Érika de Abreu Costa Brito^2,3^; Marcela Sampaio Lima^2,3^; Hianga Fayssa Fernandes Siqueira^2^; Adriane Dórea Marques^2,3^; Alex Rodrigues Moura^2,3^; Evânia Curvelo Hora^2^; Carlos Anselmo Lima^1,2,3,5^; Marceli de Oliveira Santos^4^; Mirian Carvalho de Souza^4^; Angela Maria da Silva^2,3^; Hugo Leite de Farias Brito^2,3^; Rosana Cipolotti^2,3^.

1. Aracaju Cancer Registry

2. Health Sciences Graduate Program

3. University Hospital/EBSERH/Federal University of Sergipe

4. CONPREV/Brazilian National Cancer Institute

5. Researcher, CHAMADA MS/CNPq/FAPITEC/SE/SES – No 06/2018

1. Number of incident cases (invasive) by year and age-group used to calculate age-specific, crude, and age-standardized rates.

Calculations of standard errors and confidence intervals of rates

1. Number of incident cases (in situ) by year and age-group used to calculate age-specific, crude, and age-standardized rates.

Calculations of standard errors and confidence intervals of rates

1. Number of deaths by year and age-group used to calculate age-specific, crude, and age-standardized rates.

Calculations of standard errors and confidence intervals of rates

1. Curves: Mortality; incidence, Invasive; incidence, in situ


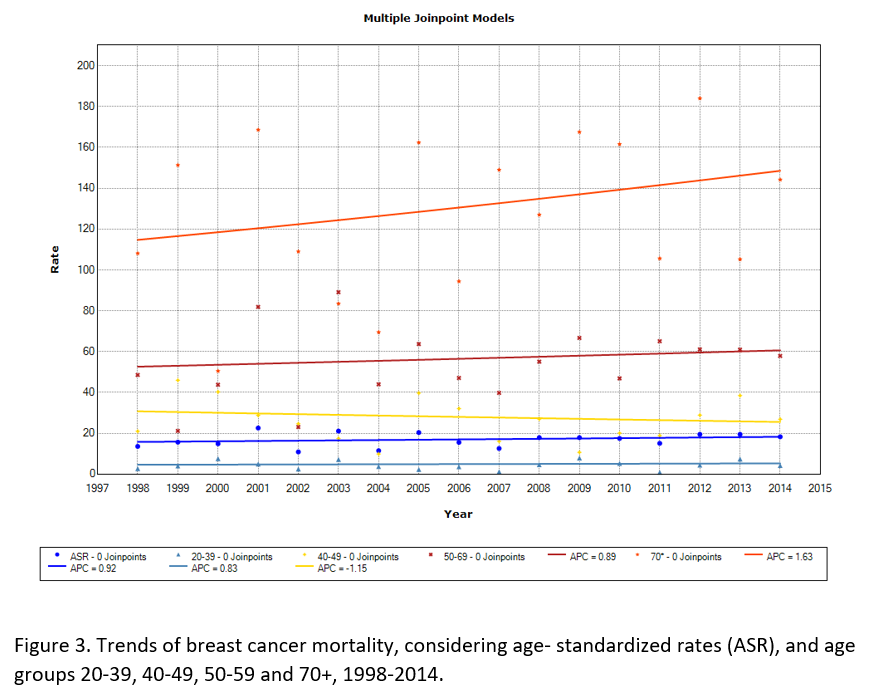

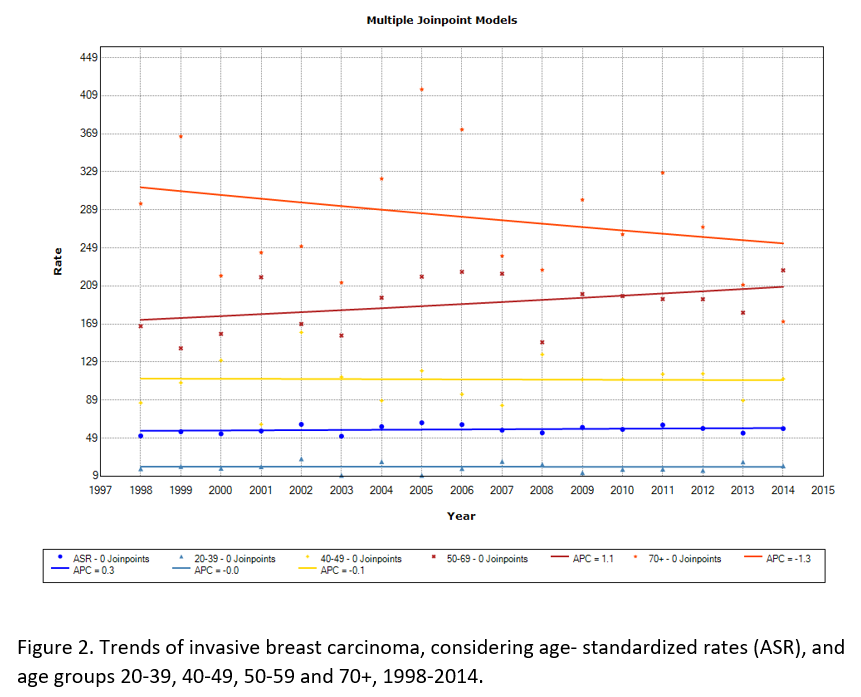

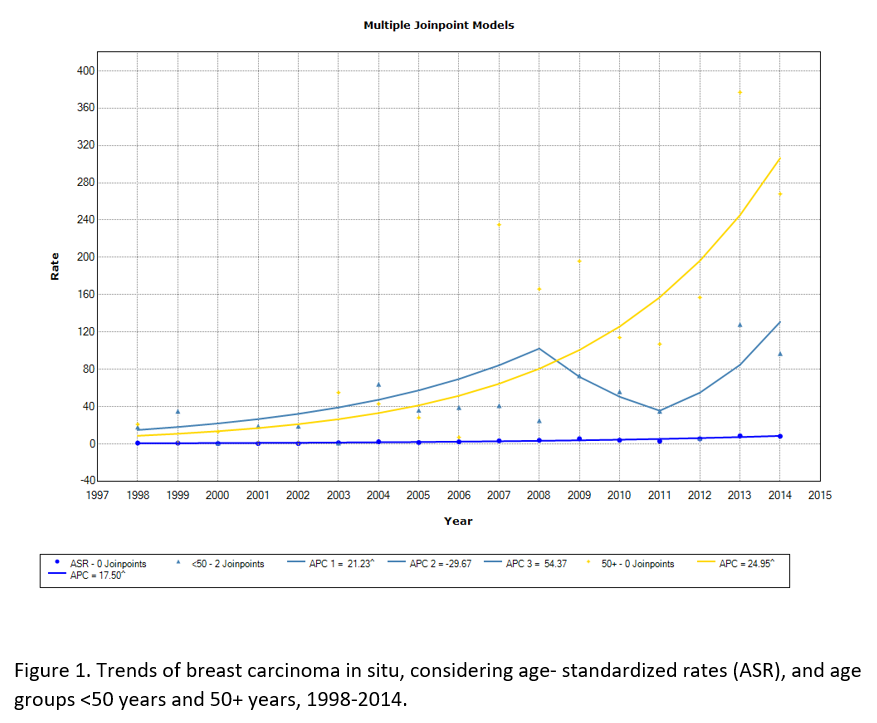


1. Trends, invasive


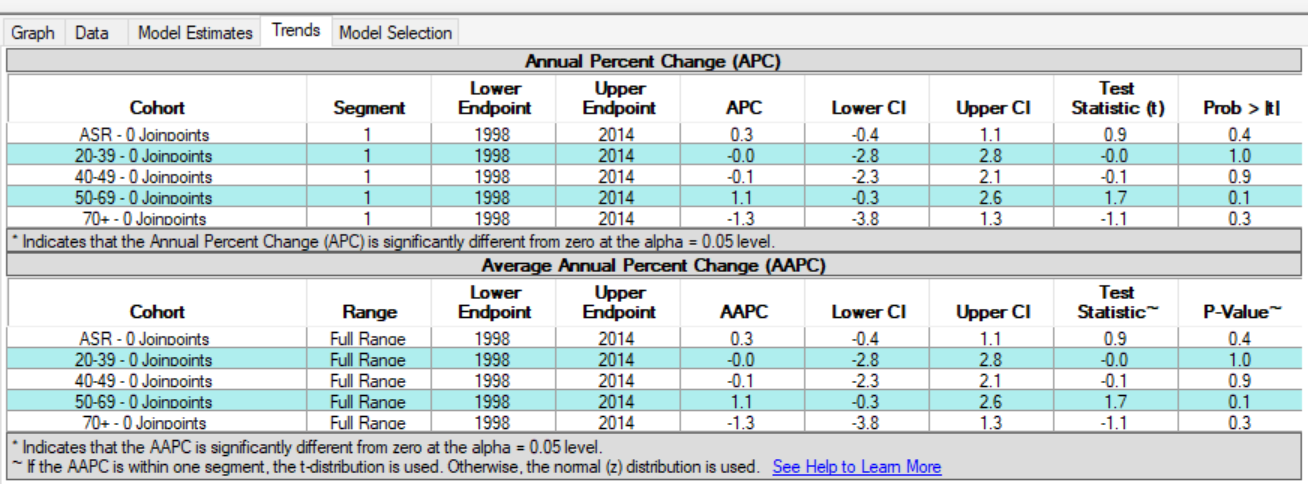


1. Trends, in situ


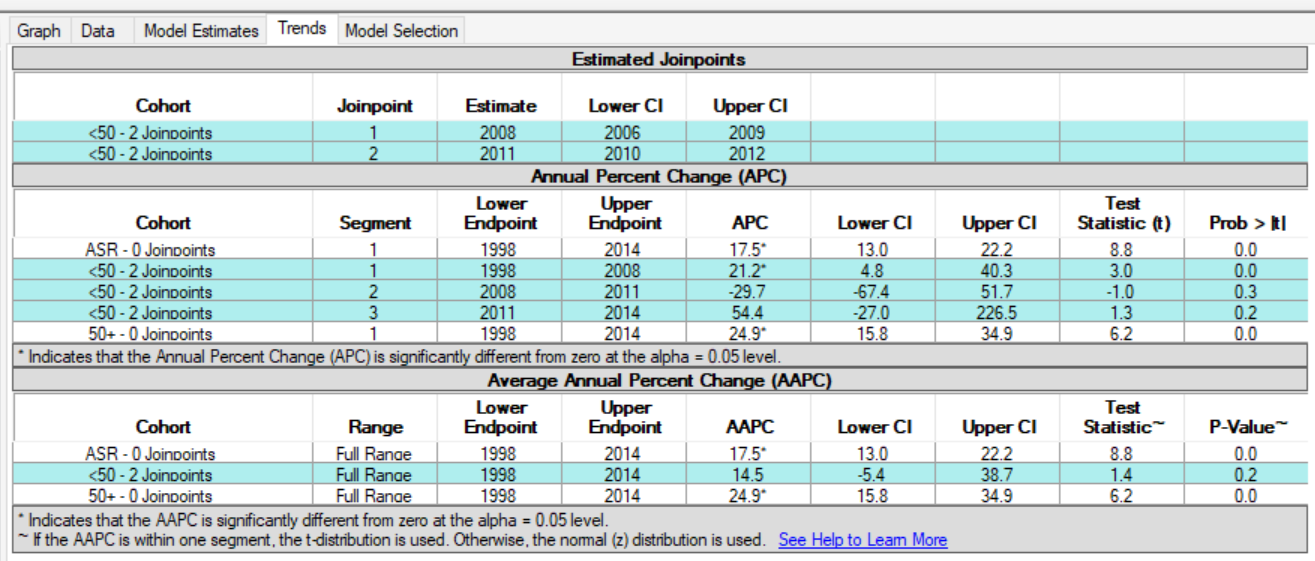


1. Trends, Mortality


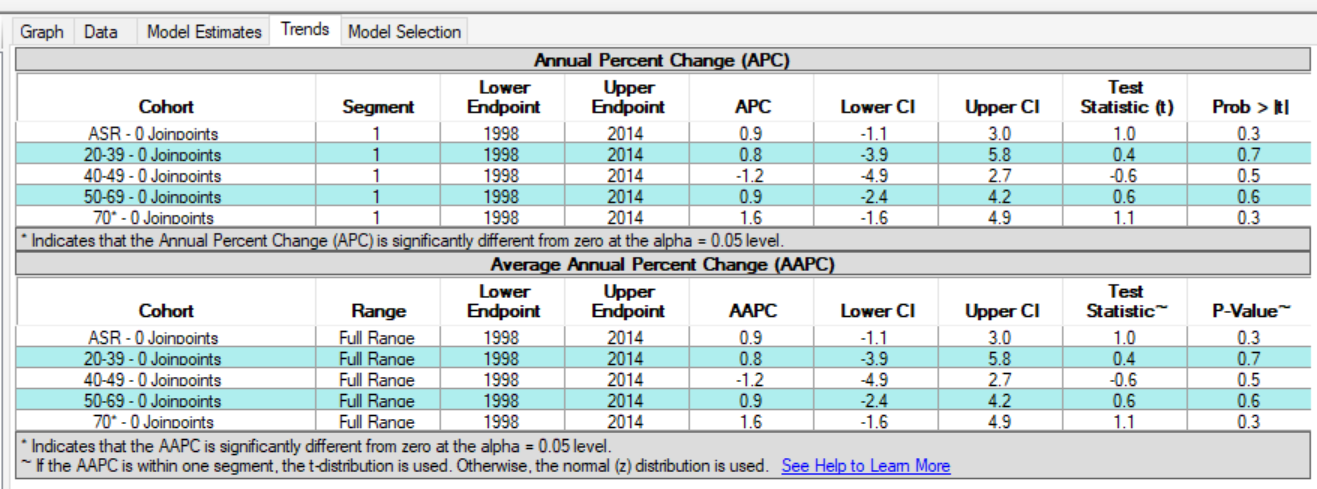

Supplement: Supplementary file 1 — Assessing trends of breast cancer and carcinoma in situ to monitor screening policies in developing settings [file 41598_2019_50504_MOESM1_ESM.docx]
